# Creating Better Collision-Free Trajectory for Robot Motion Planning by Linearly Constrained Quadratic Programming

**DOI:** 10.3389/fnbot.2021.724116

**Published:** 2021-08-09

**Authors:** Yizhou Liu, Fusheng Zha, Mantian Li, Wei Guo, Yunxin Jia, Pengfei Wang, Yajing Zang, Lining Sun

**Affiliations:** ^1^State Key Laboratory of Robotics and System, Harbin Institute of Technology, Harbin, China; ^2^Robotics Institute, Shenzhen Academy of Aerospace Technology, Shenzhen, China; ^3^Harbin Mingkuai Machinery & Electronics Co., Ltd., Shenzhen, China

**Keywords:** trajectory optimization, quadratic program, constrained motion planning, collision backtracking, robot manipulation planning

## Abstract

Many algorithms in probabilistic sampling-based motion planning have been proposed to create a path for a robot in an environment with obstacles. Due to the randomness of sampling, they can efficiently compute the collision-free paths made of segments lying in the configuration space with probabilistic completeness. However, this property also makes the trajectories have some unnecessary redundant or jerky motions, which need to be optimized. For most robotics applications, the trajectories should be short, smooth and keep away from obstacles. This paper proposes a new trajectory optimization technique which transforms a polygon collision-free path into a smooth path, and can deal with trajectories which contain various task constraints. The technique removes redundant motions by quadratic programming in the parameter space of trajectory, and converts collision avoidance conditions to linear constraints to ensure absolute safety of trajectories. Furthermore, the technique uses a projection operator to realize the optimization of trajectories which are subject to some hard kinematic constraints, like keeping a glass of water upright or coordinating operation with dual robots. The experimental results proved the feasibility and effectiveness of the proposed method, when it is compared with other trajectory optimization methods.

## 1. Introduction

Sampling-based motion planners (SBMPs), such as Probabilistic Road Maps (Kavraki et al., 1996) (PRMs) or Rapidly-exploring Random Trees (LaValle and Kuffner, 2001) (RRTs), have become the mainstream methods for solving motion planning problems in high-dimensional space because of their high efficiency and probabilistic completeness. Today, most of the cutting-edge motion planning methods like RRT^*^ (Karaman and Frazzoli, 2011), Fast Matching Tree (FMT) (Janson et al., 2013) and Stable Sparse RRT* (SST*) (Bekris et al., 2016) are inspired by SBMPs, which can ensure the final trajectory is collision-free. However, considering the service life and efficiency of equipment in practice, there are higher requirements for the trajectory quality, that is, a good trajectory should be short, smooth, dynamically feasible, and keep minimum clearance to obstacles. Therefore, it is necessary to post-process the trajectory generated by SBMPs.

**Figure 1:**
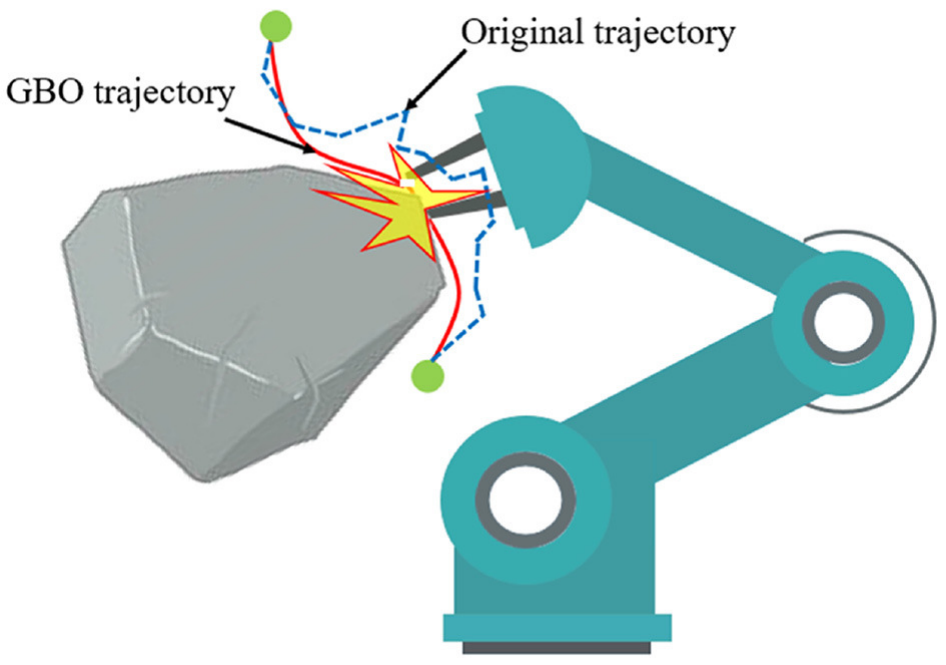
When GBO falls into local optimal, the collision-free state of trajectory may be broken. Because the weighted cost function may cause GBO to ignore obstacles term *f*_*obs*_(ξ) at the local optimal point. The blue dotted line is the original trajectory produced by SBMPs, which is collision-free, while the red line is the optimized trajectory processed by GBO.

The post-processing methods for high-dimensional space trajectories can be roughly divided into two categories. One category randomly selects two configurations along the trajectory and attempts to replace the intervening sub-path with a better path, like straight lines (Kallmann et al., 2010), Bezier curves (Yang and Sukkarieh, 2010) or B-splines (Maekawa et al., 2010), so called Short-cut. Short-cuts can shorten trajectory efficiently and easily, but they cannot provide enough flexibility in terms of generating collision-free smooth trajectories in some complicated circumstances. The other category is gradient-based optimization (GBO) methods (Ratliff et al., 2009; Kalakrishnan et al., 2011). They treat a trajectory ξ as a point in a possibly infinite-dimensional space, and use the weighted sum of the smooth cost function *f*_*smooth*_(ξ) and the obstacle cost function *f*_*obs*_(ξ) to evaluate the path quality. Thus, a motion planning problem is transformed into an iterative numerical optimization problem. There are two key issues which need to be solved in GBO: (1) Computing obstacle cost *f*_*obs*_(ξ) in an inexpensive and accurate way (2) Ensuring the trajectory is collision-free in case of unavoidable local optimal, as shown in Figure 1.

The idea of our method is to find a good balance between Short-cut and GBO. On the one hand, the method adopts the gradient descent idea of GBO to avoid the blindness and inflexibility of Short-cut, so as to obtain higher-order smoothness or to handle narrow passages. On the other hand, the method do not treat obstacle as a term of cost function, but as linear constraints of quadratic programming which are added incrementally. In this way, the local minimum problem of GBO will not break the collision-free condition. Moreover, since there is no need to quantify the collision cost, the method can avoid the time-consuming distance or penetration computation and need not perform any preprocessing of the robot or the environment. Therefore, the proposed method retains the advantages of GBO and avoids the above two difficult issues.

**Figure 2:**
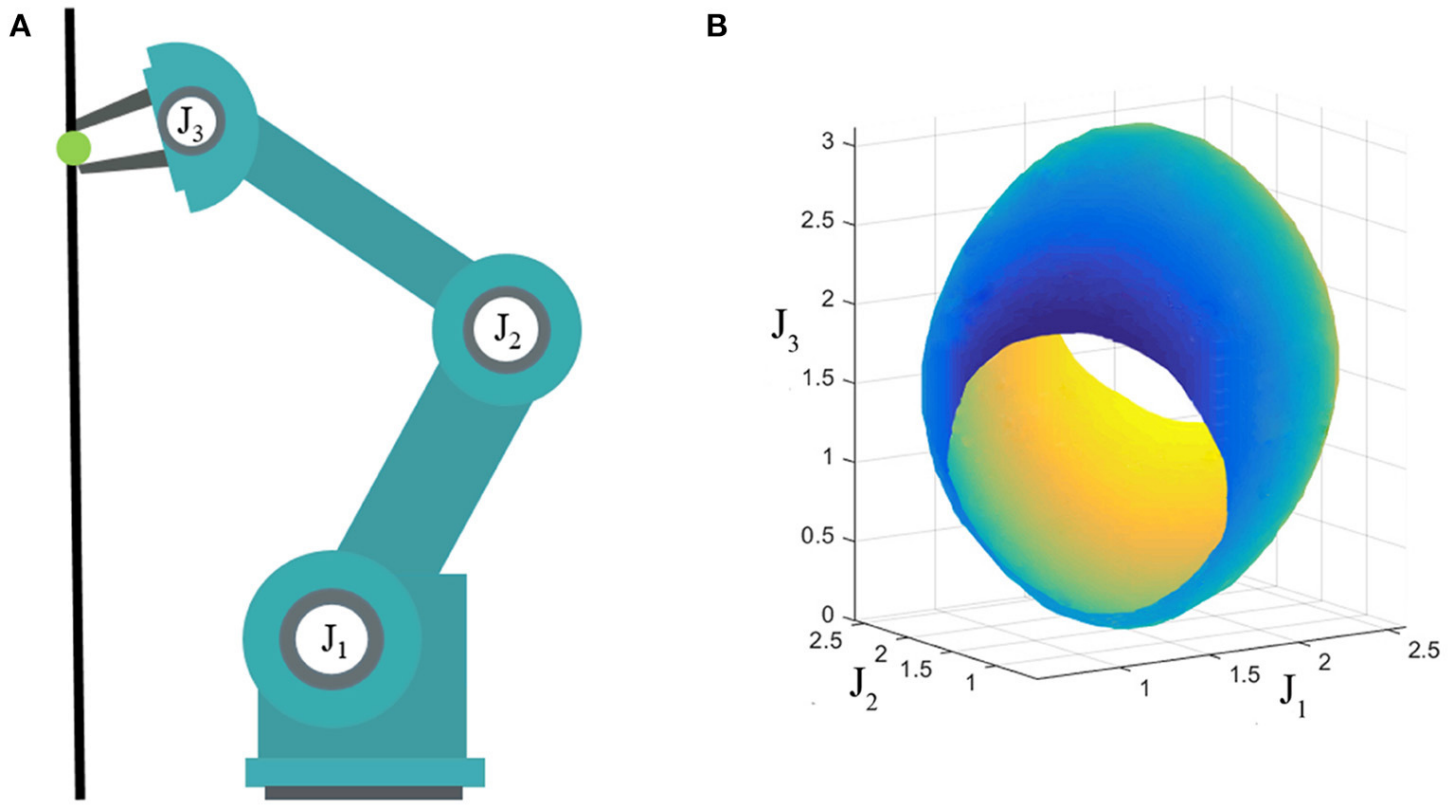
**(A)** Three degree-of-freedom (DOF) robot; **(B)** Constraint manifold. The end effector of a three-DOF robot is constrained on the black line, which means that the robot has only two-DOF in the workspace. The configurations satisfying the constraint form a two-dimensional manifold in the C-space which is shown in **(B)**.

Another important feature of our method is that it can be used to optimize the trajectories which are subject to task constraints. Task constraints are everywhere in motion planning. For instance, a robot should keep the end-effector's orientation fixed when transferring a glass filled with water, or when multiple robots form a closed kinetic chain to collaborate on a task. Due to task constraints, the feasible configurations form a low dimensional manifold in the original Configuration Space (C-space) which is nonlinear and has zero measure (Qureshi et al., 2020), as shown in Figure 2. The existing Short-cut and GBO methods will destroy the task constraints which are already satisfying. In other words, they will make the path on manifold drift away from the constraint manifold. The proposed method uses a stepwise projection technique to solve the optimization problem under task constraints, and has been proven effective with series of experiments.

The main contribution of this paper is: We propose a novel robot trajectory post process algorithm for sampling-based motion planners. (1) The method is realized by gradient-based optimization method without any distance calculation and environment models, which can effectively improving the trajectory quality and ensure the final trajectory is totally collision-free. (2) This method can effectively optimize the robot trajectory under various task constraints.

The paper is organized as follows: section Related Work summarizes the existing research related to the proposed method; section Method introduces the implementation detail and the quantitative evaluation of the proposed method; section Experiments provides a series of experimental results and analysis. In section Conclusion, conclusions are drawn and directions for future work are provided.

## 2. Related Work

Autonomous navigation and planning in complex environments are fundamental problems faced by almost all robots. Although neural network-based motion planning algorithms (Qureshi et al., 2019; Li et al., 2020; Pandey et al., 2020) have received extensive attention in recent years, SBMP still stays as the mainstream motion planning method due to its high reliability and efficiency. The trajectory generated by SBMPs is accompanied by a lot of redundant motions, which causes unnecessary jerk and detours during the operation of equipment. For the purpose of a shortened path or higher-order simplification, some methods for post-processing SBMPs trajectories are widely used, including Short-cut and GBO. The research work of the two categories will be reviewed and summarized in this section. Besides, as another main contribution of this paper, the motion planning and optimization methods under task constraints will be briefly discussed.

### 2.1. Short-Cut

Short-cut techniques are light-weight methods which can generate smooth motions in a heuristic way. Hauser and Ng-Thow-Hing (2010) propose to use a combination of lines and parabolas to replace the original segmentations, and the new motions are strictly subject to collision constraint, velocity bounds, and acceleration bounds. Inspired by this, Ran and Sidobre (2015) propose a Short-cut can satisfy higher-order bounds. Geraerts and Overmars (2007) propose Partial Shortcut, which only takes one degree-of-freedom (DOF) in consideration in each optimization step to achieve a shorter path. Because the baseline Short-cut cannot handle all redundant motions as all DOFs are interpolated simultaneously. Pan et al. (2012b) present a cubic B-splines based Short-cut which can produce an almost *C*^2^ trajectory. Different from other Short-cuts which discretize the new segments into small resolution and check each sample for collision, Pan et al. realize a fast and reliable continuous collision detection algorithm along spline trajectories. To reduce the blindness of random Short-cuts, Lamiraux et al. (2016) take trajectory length as the cost function and use quadratic programming to provide optimal iteration direction for Short-cuts. Base on this, Bogaerts et al. (2018) propose the multiple gradient descent algorithm (MGDA) algorithm to decrease path length while maintaining sensor coverage, which can be used to handle inspection tasks for industrial robots. Similar research includes (Bogaerts et al., 2019; Fu et al., 2019). However, the above methods cannot meet the requirement of higher-order smooth trajectory for real robots.

In addition to the typical Short-cut, the randomness of SBMPs provides the possibility for another lightweight post-processing method called Hybridization graphs (H-graph) (Raveh et al., 2011). For randomly extended piecewise linear paths in different runs, the quality of certain sub-paths within each path may be higher than the quality of the entire path. H-graph hybridizes the high quality sub-paths from a set of input paths to form an improved output solution. H-graph can be regarded as a generalized Short-cut, and the difference is that its shortcuts come from other solutions under the same planning problem, rather than artificially defined lines or curves. Similar research includes (Jaillet and Simeon, 2008; Luna et al., 2013).

Generally speaking, Short-cuts tend to be simple and fast, and generate high-quality collision-free trajectories in many cases. However, they may not provide enough flexibility in terms of generating collision-free smooth trajectories in the presence of narrow passages. Gradient-based optimization approaches can prevail in such cases.

### 2.2. Gradient-Based Optimization

A large number of gradient-based numerical optimization techniques have been applied in this domain. The earlier stage trajectory optimization is carried out directly in cartesian space including the artificial potential field (Warren, 1989) and the elastic band (Quinlan and Khatib, 2002). They treat the trajectory as a physical system, and simulate obstacle areas as repulsion or pressure to generate more natural trajectories. Optimization under cartesian space has shown to be effective and fast in simple scenarios. However, the approximation accuracy of the physical field to the obstacle space is limited, making these methods unreliable in slightly complicated scenarios. In order to obtain more flexibility, scholars try to optimize trajectories in the parameter space.

CHOMP (Covariant Hamiltonian Optimization for Motion Planning) (Ratliff et al., 2009) laid the basic framework for the recent GBO algorithms. The optimization objective *U*[ξ] is formulated as a function of the trajectory function ξ, which makes the trajectory cost invariant to time parameterization of the trajectory, so that the optimization process is performed in the parameter space of the trajectory. CHOMP uses gradient information to update the candidate trajectory, which makes it easily get stuck in the local minima. To solve the problem, Kalakrishnan et al. (2011) propose a stochastic optimization framework called STOMP (Stochastic Trajectory Optimization for Motion Planning). The algorithm uses a series of noisy trajectories to explore the space around the original trajectory to avoid derivative, and uses a EM-like (Expectation Maximum) method to generate a better trajectory. On the basis of the above, Incremental Trajectory Optimization for Motion Planning (ITOMP) (Park et al., 2012), Multigrid CHOMP (He et al., 2013) and T-CHOMP (Byravan et al., 2014) make various improvements for dynamic scenarios, path quality and computational efficiency. However, these GBOs have a common feature being that they use pre-computed signed distance field for calculating obstacle term to reduce the online computational overheads. On the one hand, it is difficult for the distance field to accurately describe complex obstacles, which leads to trajectory safety issues. On the other hand, robot or environment changes will lead to failure of the distance field, which limits the flexibility of the methods. Some other GBOs like Trajectory Optimizer (TrajOpt) (Schulman et al., 2013) need not build a distance field off-line, but calculate the nearest obstacle distances at each discrete time of the trajectory vector. It is also a burden for high-dimensional robots or complex scenarios.

The above GBOs can be used as post-process steps, but they are essentially motion planning methods. Therefore, the initial trajectory can be arbitrary in theory, although some inappropriate initial trajectories may lead to bad performance of the method. In contrast, our method requires the initial trajectory to be totally collision-free. Besides, the proposed method need not build any approximate model offline, or compute any distance or penetration online.

### 2.3. Constrained Motion Planning and Optimization

Same as obstacle avoidance, constraints involving the pose of a robot's end-effectors are common constraints in motion planning, such as carrying a cup of water, opening a door or kinematic loop-closure. The allowed configurations of the robot form a lower-dimensional manifolds in the C-Space which is nonlinear, zero measure and non-analytical description. Effectively sampling and exploring on constrained manifolds is the key to solving the problem.

The methods for solving above problem can be divided into four categories: Relaxation (Bialkowski et al., 2013; Bonilla et al., 2017), Tangent-space (Kim et al., 2016), Atlas (Bordalba et al., 2017), and Projection (Stilman, 2007; Berenson et al., 2011). Relaxation is to relax the manifold's surface to transform the constrained motion planning into a narrow passage problem. Tangent-space and Atlas use the piecewise linear approximation of manifolds to describe the constraints, but are easy to break down in some highly curved regions. In contrast, Projection uses Jacobian pseudo inverse based Gauss-Newton iteration to find feasible configurations, which is accurate, stable and easy to implement. Kingston's review (Kingston et al., 2018) summarizes and compares the four types of methods in detail. Besides, Kingston et al. propose Implicit Manifold Configuration Space (IMACS) (Kingston et al., 2019) recently which decouples constraint satisfaction strategies like relaxation, projection and Atlas from the choice of underlying motion planners. Their framework allows a broad range of SBMPs to operate under kinematic constraints.

Planning with neural network has became popular in recent years. A most recent work called Constraint Motion Planning Networks (CoMPNet) (Berenson et al., 2009), which is developed from Motion Planning Network (MPNet) (Qureshi et al., 2019), leverages past planning experience for learning a deep neural model and generates samples on the implicit manifolds. Except for sampling technique, CoMPNet performs same path search process on manifolds as Constrained Bi-direction Rapidly Exploring Tree (CBiRRT) (Berenson et al., 2011). Equality Constraint Manifold Neural Network (ECoMaNN) (Fernández et al., 2020) shares similar ideas with CoMPNet, which uses Variational Auto Encoders (VAE) (Kingma and Welling, 2014) to learn implicit constraint manifolds from data and generates feasible samples for SBMPs' framework. Reinforcement learning based motion planning is another fast-growing method (Bing et al., 2019). Bing et al. (2020) proposed an IRL-based controller based on the adversarial inverse reinforcement learning (AIRL) algorithm to realize the energy-efficient and damage-recovery slithering gait of a snake-like robot. What's more, some neuro-based motion planning methods inspired by the biological intelligence of living creatures have also been proved to be feasible and efficient (Bing et al., 2018).

The above methods can solve the problem of local planning and sampling on the manifold, but the path still needs to be optimized. Dragan et al. (2011) extend CHOMP to constrained CHOMP, which uses the method of Lagrange multipliers to set up a gradient descent problem to optimize trajectories under constraints. Based on this, He et al. (2013) propose Multigrid CHOMP to improve the runtime of constrained CHOMP without significantly reducing optimality. However, as mentioned above, when the CHOMP-like methods inevitably get stuck in local minima, the collision-free condition of the trajectory will be broken, so will the task constraints. In our framework, both obstacle avoidance and task constraints will be strictly satisfied.

## 3. Method

In this section, we will deduce the mathematical principle and updating law of the trajectory optimization strategy. As a basis for collision-free optimization, the collision backtracking mechanism will be introduced in detail. Then we explore the feasibility of using stepwise projection technique to apply the proposed method to constrained motion optimization. Finally, to quantify the trajectory quality in subsequent experiments, two evaluation indicators are briefly introduced.

### 3.1. Mathematical Principle

The nomenclature is shown in Table 1. Like most GBOs, the method treats the trajectory as a point in an infinite dimensional parameter space. The number of robot joints is *n*, which can be expressed as (*J*_1_, *J*_2_, ⋯ , *J*_*n*_). The configuration space of the robot is *Q* ⊂ ℝ^*n*^. The subset of *Q* composed of collision-free configurations is collision-free configuration space *Q*_*col*_*free*_. The path points number on the trajectory is *m*, so the robot trajectory can be expressed as ξ ∈ ℝ^*mn*^:

(1) $$\begin{array}{l} \xi =\left(\underset{J_{1}}{\underset{︸}{q_{1}^{0},q_{1}^{1},\ldots ,q_{1}^{m},}}\underset{J_{2}}{\underset{︸}{q_{2}^{0},q_{2}^{1},\ldots ,q_{2}^{m},}}\ldots ,\underset{J_{n}}{\underset{︸}{q_{n}^{0},q_{n}^{1},\ldots ,q_{n}^{m},}}\right) \end{array}$$

**Table 1:** Nomenclature.

*Q*	Robot configuration sapce
*Q*_*col*_*free*_	Collision-free configuration sapce
ξ	Robot trajectory
*U*(ξ)	The optimization object funtion
*g*_*k*_	The gradient of Optimization
α_*k*_	The update rate
***K***	The finite differential matrix
***u***	The unit vector
***J***	The Jacobian matrix
***J*^+^**	The Jacobian pseudo inverse matrix
***P***	The Points in cartesian space
***q***	The Points in robot configuration sapce
***C***	The linear equality constraints
***S***	The linear inequality constraints
***H***	The Hessian matrix
***B*_*t*_**	The bounding box of task constraints
$M_{C}$	The constraint manifold
*T*_*E*_	The execution time of trajectory
*R*	The smoothness ratio
*P*^+^, *L*^+^, *P*^−^, *L*^+^	The motion primitives

Because the method treats collision avoidance and task constraints as hard constraints rather than optimization terms, the cost function only contains the smooth term *f*_*smooth*_(ξ) to measure dynamical quantities across the trajectory. *f*_*smooth*_(ξ) can be precisely computed by a sum of squared derivatives.

For each joint's trajectory ξ_*i*_, *i* ∈ {1, 2, …, *n*}, a finite differential matrix **K** can be constructed as:

(2) $$\begin{array}{l} \text{K}=\left[\begin{array}{ccccccc} 1 & 0 & 0 & \; & 0 & 0 & 0\\ -2 & 1 & 0 & \cdots & 0 & 0 & 0\\ 1 & -2 & 1 & \; & 0 & 0 & 0\\ \; & \vdots & \; & \ddots & \; & \vdots & \;\\ 0 & 0 & 0 & \; & 1 & -2 & 1\\ 0 & 0 & 0 & \cdots & 0 & 1 & -2\\ 0 & 0 & 0 & \; & 0 & 0 & 1 \end{array}\right]⊗I_{m\times m} \end{array}$$

which will make:

(3) $$\begin{array}{l} \ddot{\xi_{i}}=\text{K}\xi_{i} \end{array}$$

(4) $$\begin{array}{l} {\ddot{\xi_{i}}}^{\text{T}}\ddot{\xi_{i}}=\xi_{i}^{\text{T}}\left({\text{K}}^{\text{T}}\text{K}\right)\xi_{i}=\xi_{i}^{\text{T}}\text{R}\xi_{i} \end{array}$$

where **R** = **K**^T^**K**, and $\xi_{i}^{\text{T}}\text{R}\xi_{i}$ represents the sum of squared accelerations along the *i*th joint's trajectory.

In order to calculate the weighted sum of the smooth cost of all joints' trajectories, we construct a positive Hessian matrix **H**:

(5) $$\begin{array}{l} \text{H}=\left[\begin{array}{cccc} w_{1}\text{R} & 0 & \cdots & 0\\ 0 & w_{2}\text{R} & \cdots & 0\\ \vdots & \vdots & \ddots & \vdots \\ 0 & 0 & \cdots & w_{n}\text{R} \end{array}\right] \end{array}$$

where *w*_*i*_ is the weight of the joint *J*_*i*_.

In this way, the optimization object can be formulated as:

(6) $$\begin{array}{l} \;U\left(\xi \right)=\frac{1}{2}\overset{n}{\underset{i=1}{\sum }}w_{i}||\xi_{i}|{|}_{\text{R}}^{2}=\frac{1}{2}\xi^{\text{T}}\text{H}\xi \end{array}$$

Through a first order Taylor expansion, the optimization object can be expressed as:

(7) $$\begin{array}{l} U\left(\xi \right)\approx U\left(\xi_{k}\right)+g_{k}^{\text{T}}\left(\xi -\xi_{k}\right) \end{array}$$

where *g*_*k*_ is the gradient of the optimization object at ξ_*k*_:

(8) $$\begin{array}{l} g_{k}=\nabla \left(U\left(\xi_{k}\right)\right)=\nabla \left(\frac{1}{2}{\xi_{k}}^{\text{T}}\text{H}\xi_{k}\right)=\text{H}\xi_{k} \end{array}$$

Further, the update rule after adding regular term can be expressed as:

(9) $$\begin{array}{l} \xi_{k+1}=\arg\underset{\xi }{\min}\left\{U\left(\xi_{k}\right)+g_{k}^{\text{T}}\left(\xi -\xi_{k}\right)+\frac{\lambda }{2}||\xi -\xi_{k}|{|}_{M}^{2}\right\} \end{array}$$

Let Δξ = ξ−ξ_*k*_, then:

(10) $$\begin{array}{l} \Delta \xi =\arg\underset{\Delta \xi }{\min}\left\{U\left(\xi_{k}\right)+g_{k}^{\text{T}}\Delta \xi +\frac{\lambda }{2}||\Delta \xi |{|}_{\text{M}}^{2}\right\} \end{array}$$

where the notation $||\Delta \xi |{|}_{M}^{2}$ denotes the norm of displacement between the current trajectory and the updated trajectory with respect to the Riemannian metric *M*. λ is a normalizing factor to balance the trajectory smoothness and the updating of step length.

Therefore, the trajectory updating process without constraints can be expressed as:

(11) $$\begin{array}{l} xi_{k+1}=\xi_{k}+\alpha_{k}\Delta \xi_{k} \end{array}$$

where α_*k*_ is the update rate of the *k*th iteration.

So far, we have deduced the optimization process without considering collision avoidance and task constraints.

### 3.2. Collision Backtrack

The initial trajectory ξ_0_ which needs to be post process is totally collision-free, and most of SBMPs can guarantee this:

(12) $$\begin{array}{l} \text{q}\in Q_{col\text{\_}free},\;\forall \text{q}\in \xi_{0} \end{array}$$

But the changes to the trajectory caused by the optimization process may lead to violation of the collision-free state. So we propose the collision backtrack mechanism to convert the collision avoidance condition into linear constraints of quadratic programming.

**Figure 3:**
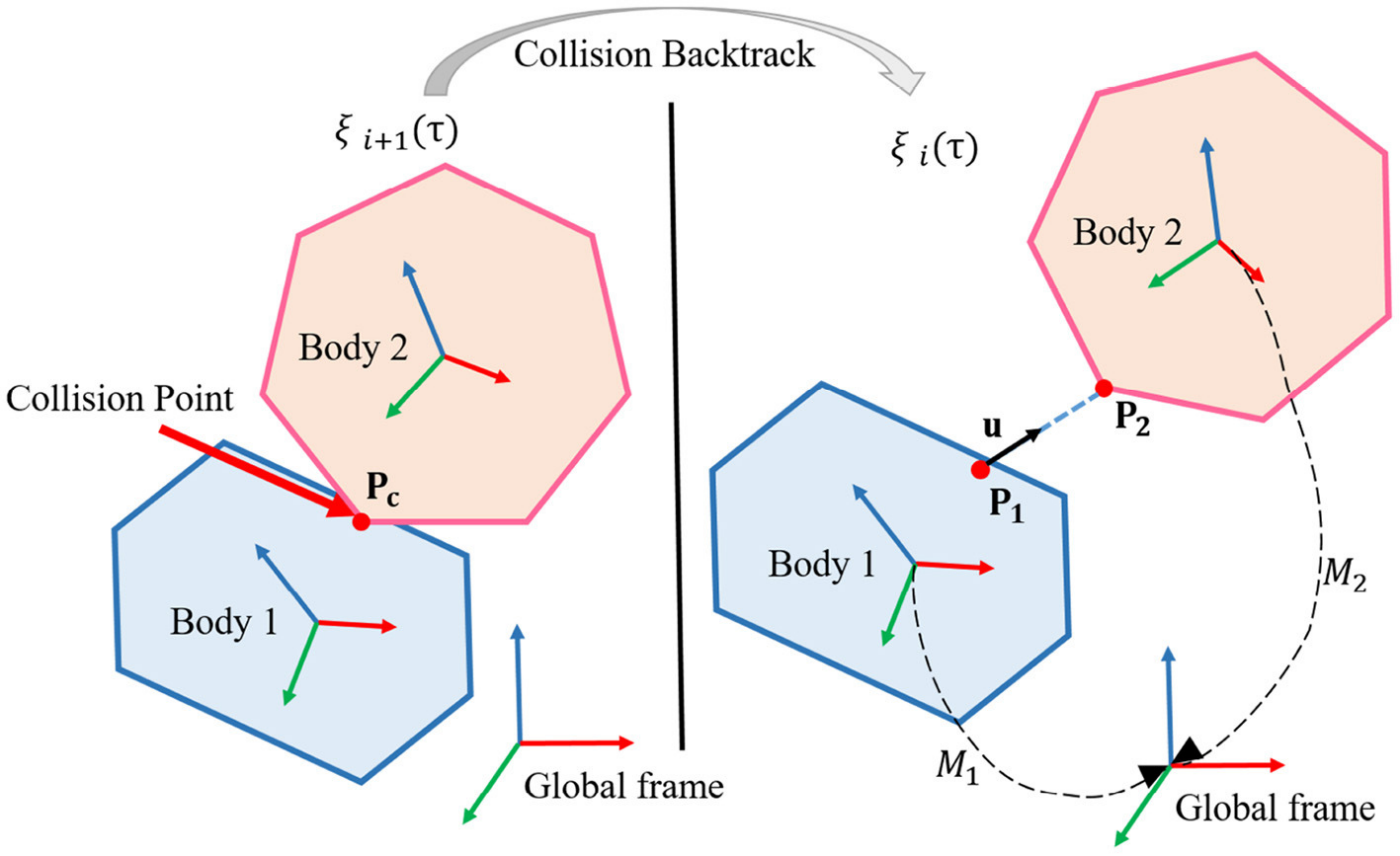
Diagram for collision backtrack.

Assume that after *i* iteration, the trajectory is still collision-free. In *i* + 1 iteration, a collision occurs at τ[τ ∈ (0, 1)] moment, and the collision points on the two rigid bodies are **P**_1_ and **P**_2_, respectively. We backtrack to the previous collision-free trajectory ξ_*i*_ at τ moment, and the two states are shown in Figure 3.

The unit vector between two points **P**_1_, **P**_2_ is defined as **u**:

(13) $$\begin{array}{l} \text{u}=\frac{M_{2}^{1}\left(\xi_{i}\left(\tau \right)\right){\text{P}}_{2}-{\text{P}}_{1}}{\left||M_{2}^{1}\left(\xi_{i}\left(\tau \right)\right){\text{P}}_{2}-{\text{P}}_{1}|\right|} \end{array}$$

where $M_{2}^{1}\left(q\right)=M_{1}{\left(q\right)}^{-1}M_{2}\left(q\right)$, is the transformation matrix between the two rigid bodies when the robot configuration is *q*, which can be solved by positive kinematic.

**Figure 4:**
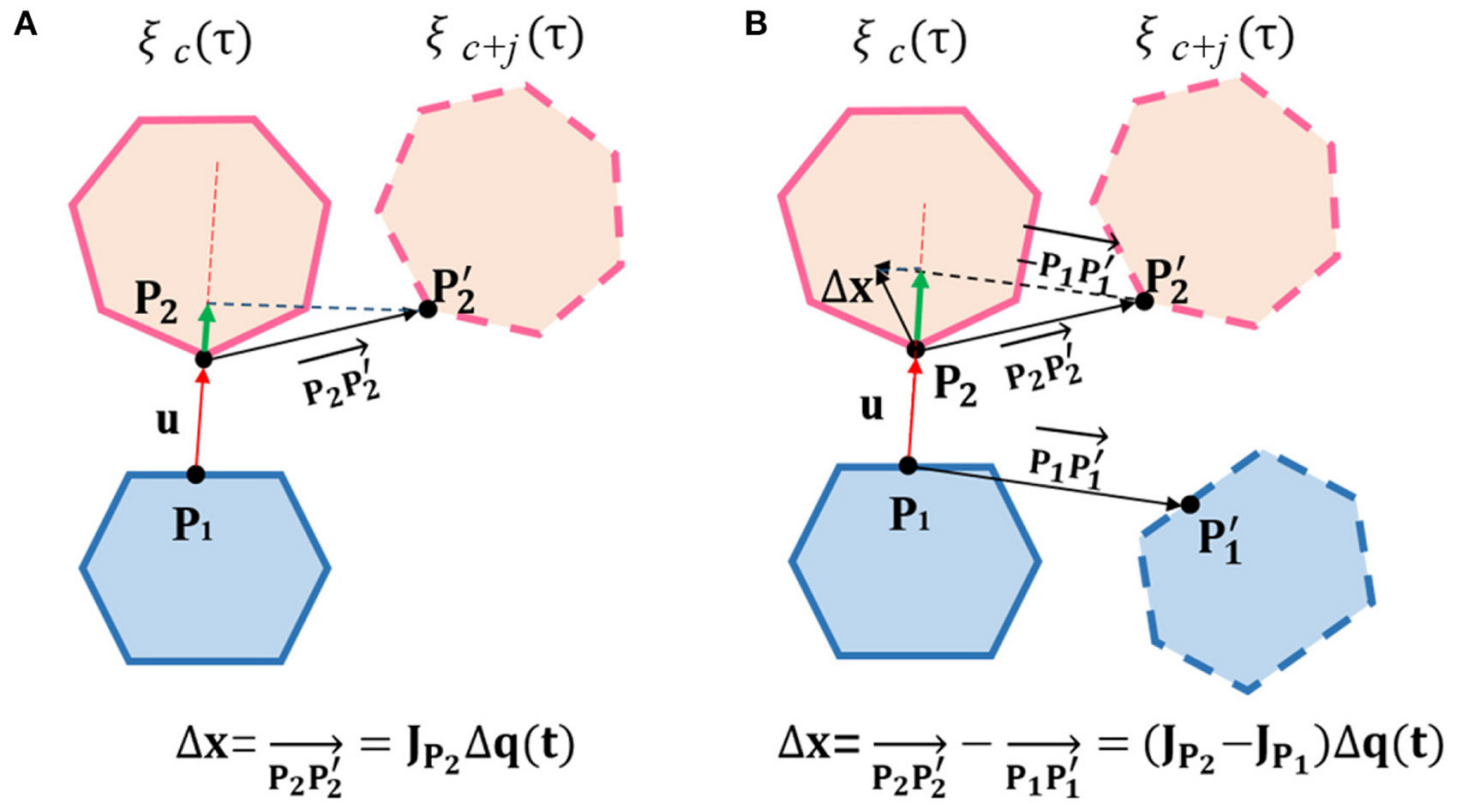
**(A)** One rigid body is on the robot, while the other is on a static obstacle; **(B)** Both rigid bodies are on the robot. Diagram for linear constraints calculation.Diagram for linear constraints calculation. ξ_*c*_(τ) is the state after collision backtracking. ξ_*c*+*j*_(τ) is the state after *c* + *j* iteration in the future. Δ*x* is the relative position change between the collision points, and the green line represents Δ*x* projection on *u*.

In order to ensure there is no more collision at this position in future optimization, we should forbid **P**_1_ and **P**_2_ to move toward each other. Mathematically, the projection of the relative position change vector Δ**x** of the two points on **u** should be ≥0. As shown in Figure 4, we discuss the two situations according to the location of the collision points.

If one of the collision rigid bodies is on the robot, the other is on a static obstacle:

(14) $$\begin{array}{l} {\text{u}}^{\text{T}}{\text{J}}_{{\text{P}}_{2}}\Delta {\text{q}}_{\tau }={\text{u}}^{\text{T}}{\text{J}}_{{\text{P}}_{2}}{\text{X}}_{\tau }\left(\xi_{c+j}-\xi_{c}\right)\geq 0 \end{array}$$

where **J**_**P**_**2**__ is a 3 × *n* Jacobian matrix, and Δ**q**_τ_ is the joints increment of the robot between ξ_*c*_(τ) and ξ_*c*+*j*_(τ).

To extract the robot configuration at τ moment, a *n* × *mn* sparse matrix **X**_**τ**_ is constructed. Since the path is a combination of piecewise linear sub-paths, we can find the two path points **q**^*k*^ and **q**^*k*+1^ adjacent to τ in the time dimension. There exists a β ∈ [0, 1] that enables ξ(τ) to be written in linear combination of two path points:

(15) $$\begin{array}{l} \xi \left(\tau \right)={\text{q}}^{k}+\beta \left({\text{q}}^{k+1}-{\text{q}}^{k}\right)=\left(1-\beta \right){\text{q}}^{k}+\beta {\text{q}}^{k+1},\beta \in \left[0,1\right] \end{array}$$

(16) $$\begin{array}{l} \text{B}={\left[\begin{array}{c} \underset{k}{\underset{︸}{0,\cdots \;,0,1-\beta ,}}\beta ,0,\cdots \;,0 \end{array}\right]}_{1\times m} \end{array}$$

For every joint of the robot:

(17) $$\begin{array}{l} {\text{X}}_{\tau }={\left[\begin{array}{cccc} \text{B} & 0 & \cdots & 0\\ 0 & \text{B} & \cdots & 0\\ \vdots & \vdots & \ddots & \vdots \\ 0 & 0 & \cdots & \text{B} \end{array}\right]}_{n\times mn} \end{array}$$

Similarly, if the two collision rigid bodies are both on robot, the linear constraint can be expressed as:

(18) $$\begin{array}{l} {\text{u}}^{\text{T}}\left({\text{J}}_{{\text{P}}_{2}}-{\text{J}}_{{\text{P}}_{1}}\right)\Delta {\text{q}}_{\tau }={\text{u}}^{\text{T}}\left({\text{J}}_{{\text{P}}_{2}}-{\text{J}}_{{\text{P}}_{1}}\right){\text{X}}_{\tau }\left(\xi_{c+j}-\xi_{c}\right)\geq 0 \end{array}$$

Extract the known term of the Equation (14) and (18):

(19) $$\Phi =\{\begin{array}{ll} u^{\text{T}}(J_{P_{2}}-J_{P_{1}})X_{\tau }, & P_{1},P_{2}\text{ both on robot}\\ u^{\text{T}}J_{P_{2}}X_{\tau }, & P_{2}\text{ on robot} \end{array}$$

Then the linear constraints for quadratic programming can be expressed as:

(20) $$\begin{array}{l} \text{C}=\left[\begin{array}{c} \Phi_{1}\\ \Phi_{2}\\ \vdots \\ \Phi_{k} \end{array}\right]\Rightarrow \text{C}\Delta \xi \geq 0 \end{array}$$

In addition to collision avoidance constraints, the start and ending points of the trajectory should be fixed. We use matrix **D** to extract the start and ending points of the strajectory:

(21) $$\begin{array}{l} \text{D}={\left[\begin{array}{ccccccc} 1.0, & 0, & 0, & \cdots \;, & 0, & 0, & 1.0 \end{array}\right]}_{1\times m} \end{array}$$

(22) $$\begin{array}{l} \text{S}={\left[\begin{array}{cccc} \text{D} & 0 & \cdots & 0\\ 0 & \text{D} & \cdots & 0\\ \vdots & \vdots & \ddots & \vdots \\ 0 & 0 & \cdots & \text{D} \end{array}\right]}_{n\times mn} \end{array}$$

Then the constraint condition can be expressed as:

(23) $$\begin{array}{l} \text{S}\Delta \xi =0 \end{array}$$

Ignoring the constant terms of Equation (10), and combining the constraints of Equations (20, 23), the linear constrained Quadratic programming (QP) problem for trajectory optimization can be formulated as following:

(24) $$\begin{array}{l} \begin{array}{c} \underset{\Delta \xi }{\min}\;\frac{\lambda }{2}\Delta \xi^{\text{T}}\text{M}\Delta \xi +g^{\text{T}}\Delta \xi \\ \;\text{C}\Delta \xi \geq 0\\ \;\text{S}\Delta \xi =0. \end{array} \end{array}$$

### 3.3. Algorithm

In this section, the process of trajectory optimization by linear constrained quadratic programming (LCQP) will be described in the form of pseudo codes.

As shown in Algorithm 1, the input of the algorithm is a collision-free trajectory generated by SBMPs. The output is the optimized trajectory by LCQP.

**Algorithm 1:**
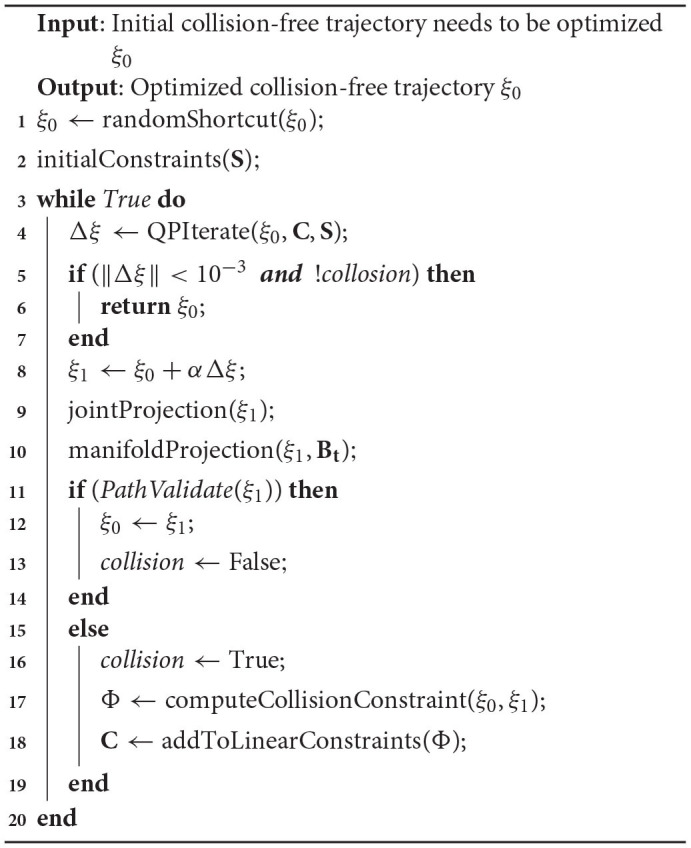
Quadratic Program-based motion trajectory postprocess.

Before the gradient-based optimization, We use randomShortcut function to remove redundant motions, which will reduce the dimensionality of the trajectory parameter space and simplify the subsequent optimization problem. But for the trajectories with task constraints, this will not be performed. Because Short-cut will greatly reduce the resolution of the trajectory, which makes the constraints unsatisfactory in some local positions. Then the boundary conditions at both ends of the trajectory are initialed to linear constraints **S**.

Lines 3 to 20 are the process of trajectory optimization. Based on the current linear constraints **C** and **S**, the incremental of the trajectory Δξ can be calculated by the LCQP which is formulated as Equation (24). If the incremental of trajectory is small enough and the previous trajectory ξ_0_ is totally collision-free, ξ_0_ will be returned as the optimization result. Otherwise, the candidate trajectory ξ_1_ will be updated according to Equation (11).

The robot's configuration might be outside of hard joint limits after the update. To ensure the trajectory satisfies the joint limits, we use the CHOMP's technique to handle joint limits by smoothly projecting joint violations. These processes are realized by jointProjection funtion at Line 9. To ensure the trajectory satisfies the task constraints, we use manifoldProjection to project the trajectory onto constraint manifold. This process will be discussed in detail in see section Task Constraints.

Then we perform an overall collision check on ξ_1_. If ξ_1_ is totally collision-free, ξ_0_ will be assigned to ξ_1_, and the collision flag of ξ_0_ will be set to False. The collision check module is realized by Flexible Collision Library (FCL) (Pan et al., 2012a), which detect collision by calculating whether the two model overlap.

If a collision occurs somewhere in ξ_1_, computeCollisionConstraint function is used to calculate the linear constraint Φ of the collision point. The function is realized by the collision backtracking method in section Collision Backtrack. Φ is added into linear constraints **C** to ensure there is no more collision at same position in future optimization.

### 3.4. Task Constraints

**Figure 5:**
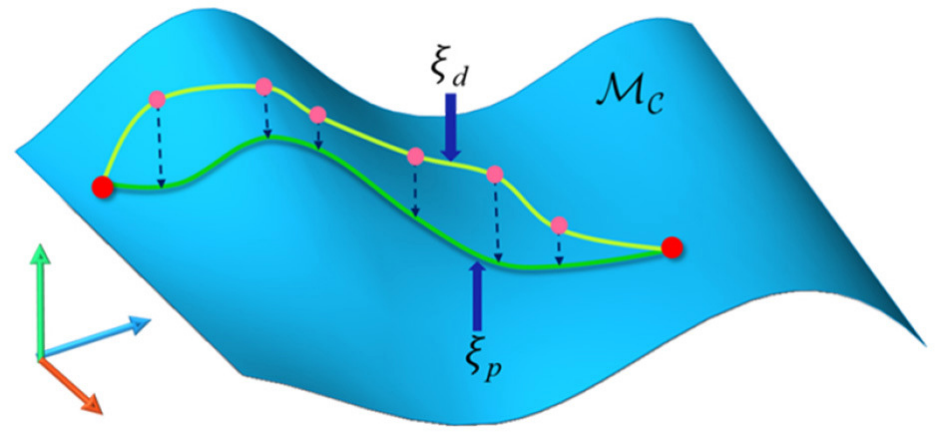
The candidate trajectory is projected onto the constraint manifold. $M_{C}$ is the constraint manifold. ξ_*d*_ is the candidate trajectory produced by LCQP iteration which may slightly violate task constraints. After projection operations, ξ_*p*_ returns to the manifold surface, which means the constraints are strictly satisfied again.

As mentioned before, the task constraints will make the feasible configurations form a lower-dimensional manifold in the ambient space. Short-cuts will cause direct damage to the task constraints which have been met. Constrained CHOMP also cannot strictly meet the task constraints. In our framework, we project every candidate trajectory produced by optimization iteration onto the constrained manifold before collision check, as shown in Figure 5. This guarantees the task constraints are strictly met from the source.

The projection onto constraint manifold is described in Algorithm 2. In order to make the trajectory satisfy the task constraint as much as possible, the original trajectory produced by SBMPs has a short step length. The trajectory's projection can be discretized into path points' projection.

**Algorithm 2:**
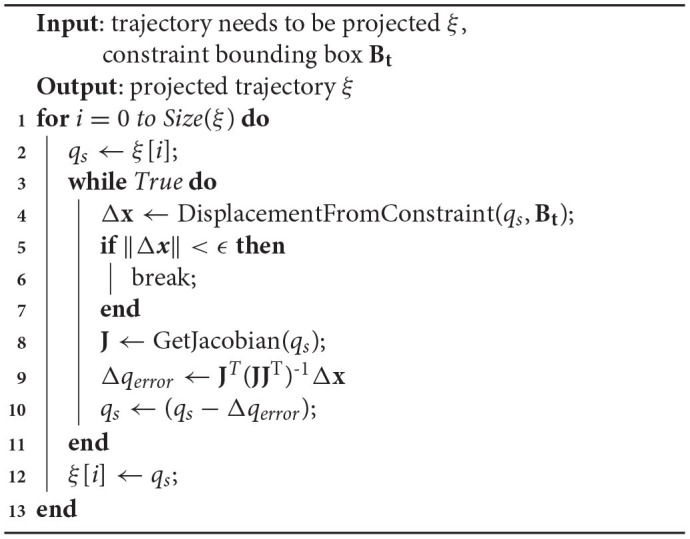
Manifoldprojection(ξ, **B**_**t**_).

The task constraints can be described as a bounding box **B**_**t**_ in cartesian space to constrain the pose of the robot's end-effector. If the end-effector's pose is in the bounding box or the distance to **B**_**t**_'s boundary is small enough, it can be considered that the task constraints are satisfied. The detailed calculation process can be found in our previous research work (Zha et al., 2018).

For configurations that do not satisfy the task constraints, we use the Gauss-Newton process based on Jacobian pseudo inverse to find nearby feasible configurations. As shown in lines 9 to 11, Jacobian pseudo inverse **J**(*q*)^+^ is used to compute the increment of joint displacement. This update can make *q*_*s*_ move toward **B**_**t**_, until the distance to **B**_**t**_ is small enough.

The above projection technique for solving task constraints has been proved to be reliable in many cases (Stilman, 2007; Berenson et al., 2011; Zha et al., 2018). This ensures the optimized trajectory always satisfies the task constraints.

### 3.5. Evaluation of Trajectory Quality

Quantitative evaluation of trajectory quality is necessary to prove the effectiveness of optimization algorithm. In this section, we will introduce two evaluation indicators to quantify trajectory quality.

Under the premise of bounded acceleration and bounded speed, the shorter the execution time is, the higher the quality of the trajectory will be. The interpolation method of line and parabola combination proposed by Hauser and Ng-Thow-Hing (2010) is used to calculate the execution time.

Assuming that each joint of the trajectory is independent, the execution time between the two path points depends on the slowest single-joint trajectory within the motion boundary. So the execution time *T*_*E*_ can be formulated as:

(25) $$\begin{array}{l} T_{E}=\underset{k}{\max}f\left(x_{1}^{k},x_{2}^{k},v_{1}^{k},v_{2}^{k},v_{max}^{k},a_{max}^{k}\right)\;k=1,2,\cdots \;,n \end{array}$$

where *x*_1_ and *x*_2_ are two path points, and *v*_1_ and *v*_2_ are velocities of the two points, respectively. The max velocity limit is *v*_*max*_. The max acceleration limit is *a*_*max*_.

Then we define four motion primitives: *P*^+^, *P*^−^, *L*^+^, *L*^−^, where *P*^+^ and *P*^−^ represent parabolas with acceleration *a*_*max*_ and −*a*_*max*_, and *L*^+^ and *L*^−^ represent a straight line with velocity of *v*_*max*_ and −*v*_*max*_, respectively. These motion primitives can be combined into four feasible motion templates: *P*^+^*P*^−^, *P*^−^*P*^+^, *P*^+^*L*^+^*P*^−^, *P*^−^*L*^−^*P*^+^. We calculate the execution time of each motion template on each sub-path and take the shortest one as the final execution time of the sub-path.

For *P*^+^*P*^−^, the inflection time *t*_*P*_ when the trajectory stops accelerating and starts decelerating can be solved by following quadratic equation:

(26) $$\begin{array}{l} a_{max}t^{2}+2v_{1}t+\left(v_{1}^{2}-v_{2}^{2}\right)/\left(2a_{max}\right)+x_{1}-x_{2}=0 \end{array}$$

The solution needs to meet the velocity boundary 0 ≤ *t* ≤ (*v*_2_ − *v*_1_)/*a*_*max*_. If the solution does not exist, the template is not valid. Otherwise, the execution time is *T* = 2*t*_*P*_ + (*v*_1_ − *v*_2_)/*a*_*max*_. Besides, *v*_1_ + *t*_*P*_*a*_*max*_ should be within the velocity boundary *v*_*max*_ too. The case of the *P*^−^*P*^+^ template is similar by negating *a*_*max*_ in the above equations.

For *P*^+^*L*^+^*P*^−^, we calculate the time duration *t*_*L*_ of the straight-line phase by the following formula:

(27) $$\begin{array}{l} t_{L}=\left(v_{2}^{2}+v_{1}^{2}-2v_{max}^{2}\right)/\left(2v_{max}a_{max}\right)+\left(x_{2}-x_{1}\right)/v_{max} \end{array}$$

where *t*_*P*1_ = (*v*_*max*_ − *v*_1_)/*a*_*max*_ is the time length of the first parabola, and *t*_*P*2_ = (*v*_2_ − *v*_*max*_)/*a*_*max*_ is the time length of the second parabola.

So the total execution time is given by

(28) $$\begin{array}{l} \;T=t_{P1}+t_{L}+t_{P2} \end{array}$$

Similarly, solution for *P*^+^*L*^+^*P*^−^ can be solved by negating *a*_*max*_ and *v*_*max*_ in the above equations.

Based on the execution time *T*_*E*_, we introduce smoothness ratio *R* which is proposed by Lau and Byl (2015). This indictor is the ratio of the execution time for resulting joint trajectories calculated according to both velocity and acceleration constraints to the execution time with only velocity limited. A smoother trajectory has a smoothness ratio closer to 1, because it does not require significant acceleration or deceleration and spends most of the trajectory with at least one joint moving at its maximum velocity. The smoothness ratio can be formulated as:

(29) $$\begin{array}{l} \;R=\frac{T_{E}}{\sum_{i\in \left\{0,1,\ldots ,N\right\}}\frac{{\text{max}}_{j}|q_{i}\left(j\right)-q_{i-1}\left(j\right)|}{v_{max}}} \end{array}$$

where *N* is the number of waypoints in the trajectory, and *q*_*i*_(*j*) corresponds to the value of joint *j* at the *i*th waypoint in the trajectory.

Execution time and smoothness ratio will be used to qualify the trajectory quality in the following experiments.

## 4. Experiments

In this section, the experiment results of the proposed method and of other trajectory optimization methods will be shown and analyzed. The SBMP algorithms used in this paper are called from Open Motion Planning Library (OMPL 1.3.1) (Sucan et al., 2012). The collision check module is supported by the Flexible Collision Library (FCL) (Pan et al., 2012a). All the experiments are implemented on an Intel Core i7–7,700 HQ 2.8 GHz laptop with 16 GB RAM. Robot Operating System (ROS) kinetic and Gazebo are used to build the simulation platform. In all experiments, we set the update ratio α = 0.2 and the task constraint tolerance ϵ = 0.1.

### 4.1. 2D Experiments

The trajectory in the robot's configuration space has high dimension, which cannot be visually observed. Therefore, we use a 2D experiment to visualize the optimization effect of the algorithm, and this process will naturally extend to multiple dimensions later.

**Figure 6:**
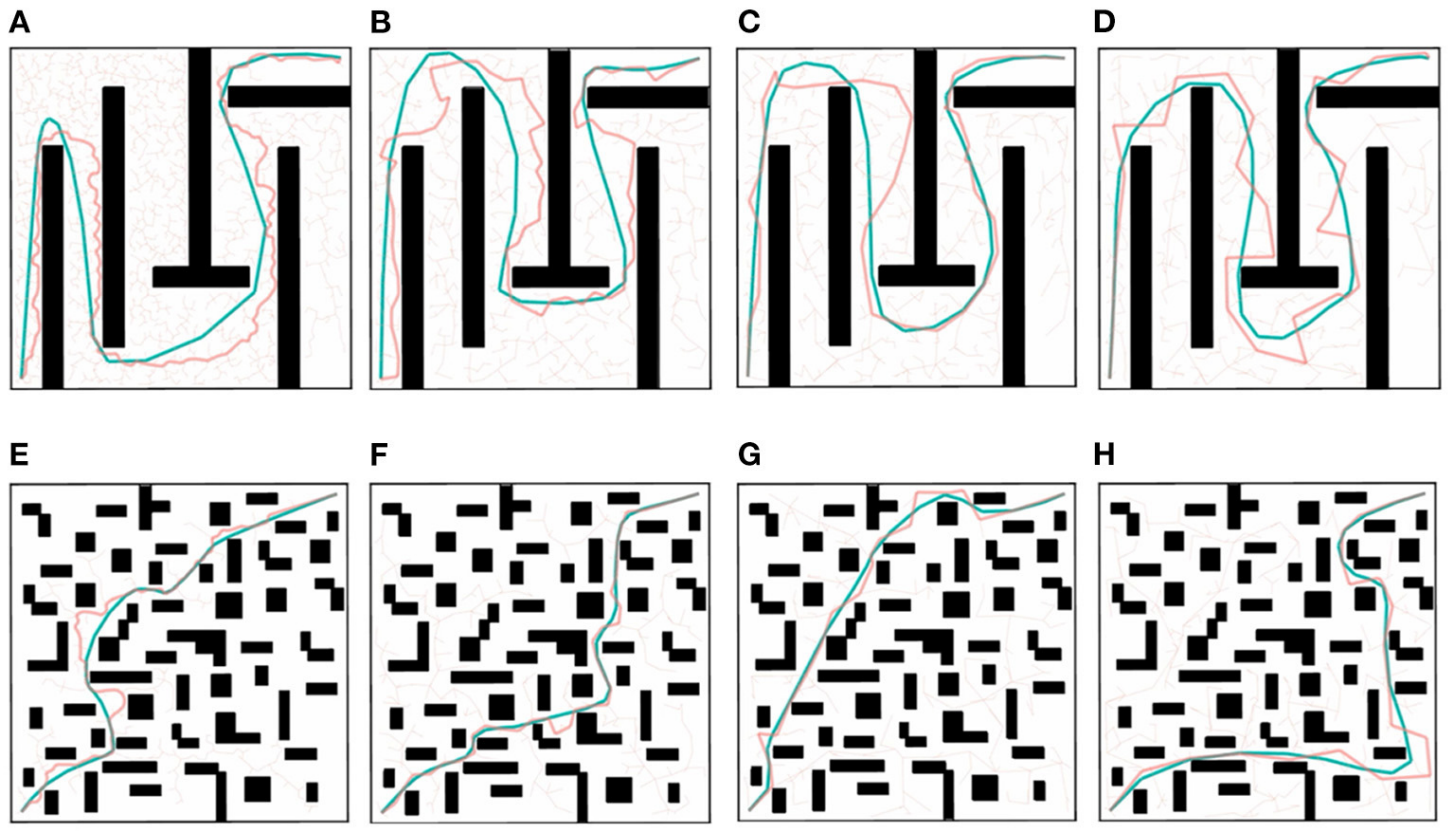
**(A)**λ = 0.02; **(B)** λ = 0.05; **(C)** λ = 0.10; **(D)** λ = 0.15; **(E)** λ = 0.02; **(F)** λ = 0.05; **(G)** λ = 0.10; **(H)** λ = 0.15. 2D experiments on a maze-like map **(A–D)** and a randomly-generated obstacles map **(E–H)** with different values of the step length λ. The red trajectory is the solution of RRT algorithm, which is a linear polygon path. The blue trajectory is the solution optimized by LCQP.

As shown in Figure 6, we use a different step length λ for the RRT algorithm, and use the proposed method to optimize the trajectory produced by RRT. The size of the 2D maps are 1 × 1 rad^2^ which means the joint limit is set as 0 rad to 1 rad. Velocity and acceleration limits used to calculate the execution time are set to 1.2 rad/s and 1.5π rad/s^2^, respectively. These values correspond to the velocity and acceleration constraints for RoboSimian (Satzinger et al., 2015). The terminal condition of the QP iteration is ||Δξ|| < 10^−3^.

Intuitively, the unnecessary jerk and motion are removed from the trajectory by LCQP, which makes the trajectory smoother and more natural. In the 2D maze-like environment of Figures 6A–D, because of the random short-cut before optimization, the trajectories with small λ have been simplified, which greatly reduces the scale of solving QP problem, and makes the optimization quality of different trajectories has good consistency. In the environment with randomly-generated obstacles, the short-cut can only remove a small amount of redundant motions, but the optimized trajectory by LCQP still has good smoothness. And it's obvious that the execution time and smoothness of the trajectories generated by the same method showed similar trends. So we guess, trajectories with better smoothness are more likely to take less execution time.

**Figure 7:**
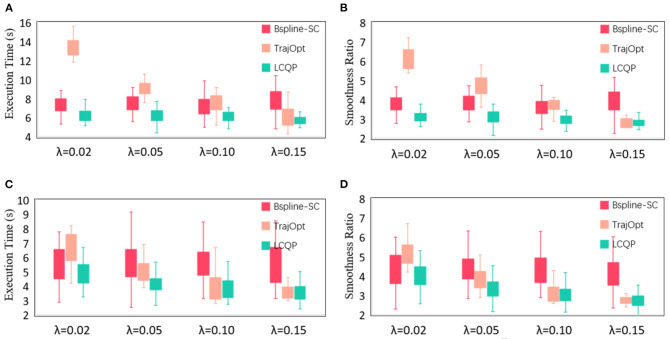
**(A)** Execution time of maze-like map; **(B)** Smoothness ratio of maze-like map; **(C)** Execution time of randomly-generated obstacles map; **(D)** Smoothness ratio of randomly-generated obstacles map. The quantitative evaluation results of three post-trajectory methods in 2D experiments.

Then, We use the other two trajectory optimization methods as comparisons to conduct quantitative experiments to verify the intuitive judgment. One of the comparison methods is B-spline Short-cut (Maekawa et al., 2010), which uses a B-spline curve to replace the redundant sub-paths. Another method is TrajOpt (Schulman et al., 2013), which uses sequential convex optimization to optimize the trajectory from any state, which is essentially a motion planning method. But it also can be used as a post-process algorithm. The results of the three optimization methods are represented by box-plots of Execution time *T*_*E*_ and Smoothness ratio *R* in Figure 7. For some items with great disparity that are inconvenient to indicate in the box plots, we display them in Table 2. Each item is obtained by 50 repeated tests.

**Table 2:** Results of the 2D-experiments.

	**Average planning time (ms)**				**Average execution time (s)**				**Average smoothness ratio**			
**λ(rad)**	**0.02**	**0.05**	**0.10**	**0.15**	**0.02**	**0.05**	**0.10**	**0.15**	**0.02**	**0.05**	**0.10**	**0.15**
**Maze-like**												
RRT Only	27.16	10.33	7.789	8.354	37.65	25.40	14.91	11.80	18.39	14.47	8.247	6.321
RRT+Bspline-SC	39.37	22.15	15.32	13.27	7.375	7.900	7.410	8.026	3.810	4.026	3.742	4.014
RRT+TrajOpt	892.4	648.3	422.1	377.0	13.29	8.974	7.532	5.988	6.182	4.750	3.663	2.832
RRT+LCQP	306.5	282.2	230.0	197.6	**6.406**	**6.263**	**6.133**	**5.690**	**3.213**	**3.081**	**3.013**	**2.798**
**Randomly-generated obstacles**												
RRT Only	6.070	3.540	4.063	5.556	19.34	14.96	12.45	9.722	13.32	9.954	7.244	4.983
RRT+Bspline-SC	22.15	18.27	16.52	16.03	5.664	5.627	5.624	5.546	4.353	4.446	4.358	4.136
RRT+TrajOpt	789.2	623.5	479.3	457.6	6.776	5.110	4.023	**3.474**	5.063	3.784	3.124	**2.726**
RRT+LCQP	195.1	132.8	105.1	98.89	**4.947**	**4.132**	**3.887**	3.576	**3.931**	**3.348**	**3.048**	2.743

The original trajectories generated by RRT have some unnecessary redundant or jerky motions, which results in long execution time and large smoothness ratio. B-spline Short-cut is efficient and easy to implement. It can remove redundant motions and improve trajectory quality in a short planning time, making it a cost-effective method. But in the case of a large step length (Figure 6D) and many narrow passages (Figures 6E–H), the effectiveness of Short-cut is inferior to GBOs because of its blindness and inflexibility. The optimization effect of TrajOpt is similar to LCPQ when λ is large. But because of the high dimension of the parameter space and necessary distance calculation, the planning time of TrajOpt is much longer than LCQP in all test items. LCQP combines the advantages of Short-cuts and GBOs, simplifying the original trajectory before QP optimization by random Short-cut, which makes the optimized trajectory much better than TrajOpt in small λ cases and has less computational overheads. Besides, compared with the B-spline Short-cut, LCQP can make good use of gradient information to converge at a lower cost to yield a better trajectory.

The above 2D experiments prove that LCQP is a cost-effective GBO method. We will extend the methods to high dimensional trajectories in the robot configuration space to verify its effectiveness.

### 4.2. Robot Trajectory Optimization

**Figure 8:**
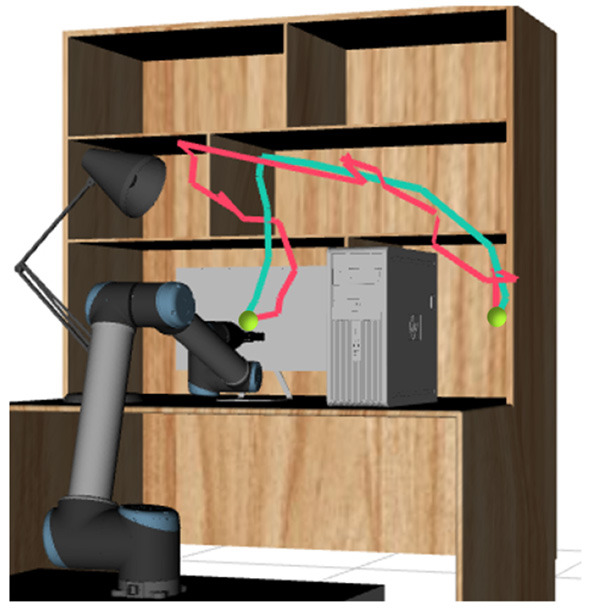
Planning scene of the robot experiment. A 6-DOF UR10 robot is used to conducted the motion task. The red trajectory of the robot end effector is the solution of RRT algorithm. The blue trajectory is the solution optimized by LCQP.

The robot experiment is conducted on a 6-DOF Universal Robot 10 (UR10) robot in a common family scene as shown in Figure 8. We use a λ value of 0.2 for the RRT algorithm, and a terminal condition ||Δξ|| < 10^−4^ for LCQP. The joint limits of the robot is −2π to 2π rad, and the velocity and acceleration boundaries are the same as 4.1.

We test the three optimization methods 50 times in the scenario of Figure 8. The high dimensional trajectories in C-space cannot be intuitively displayed, so the trajectories of the end effector which indirectly reflect the optimization effect are shown in Figure 8. The quantitative experimental results of the planning time *T*_*P*_, the execution time *T*_*E*_ and the smoothness ratio *R* are shown in Table 3.

**Table 3:** Results of the robot experiments.

	***T***_*****P*****_ **(s)**		***T***_*****E*****_ **(s)**		***R***	
	**μ**	**σ**	**μ**	**σ**	**μ**	**σ**
RRT Only	0.391	0.024	14.86	8.839	8.811	1.236
RRT + Bspline-SC	0.915	0.111	9.172	4.144	5.266	0.736
RRT + TrajOpt	4.629	0.277	4.324	1.307	3.064	0.505
RRT + LCQP	1.301	0.326	4.209	0.745	2.789	0.493

The original trajectories without any post-process have poor quality, and because of the randomness of RRT algorithm, the standard deviation of the trajectory quality is large. B-Spline Short-cut improves the original trajectory to some extent, but still cannot achieve the optimization effect of GBO methods. The proposed method LCQP does not require any obstacle distance calculations, making the planning time much shorter than TrajOpt. Since LCQP and TrajOpt are essentially quadratic programming methods, there is no big difference in the optimization effect, which is consistent with the results in the 2D experiments.

### 4.3. Optimization of Task Constrained Trajectories

Task constraints are common in robot operations, like keeping a cup of water upright, opening a door or a drawer or coordinating operation with other equipment. In this section, we will choose two typical task constraints for the experiment. As mentioned in section Related Work, Short-cuts and GBOs will destroy the task constraints which are already satisfying by making the path on manifold drift away from the constraint manifold. Therefore, B-spline Short-cut and TrajOpt are no longer applicable in this section.

**Figure 9:**
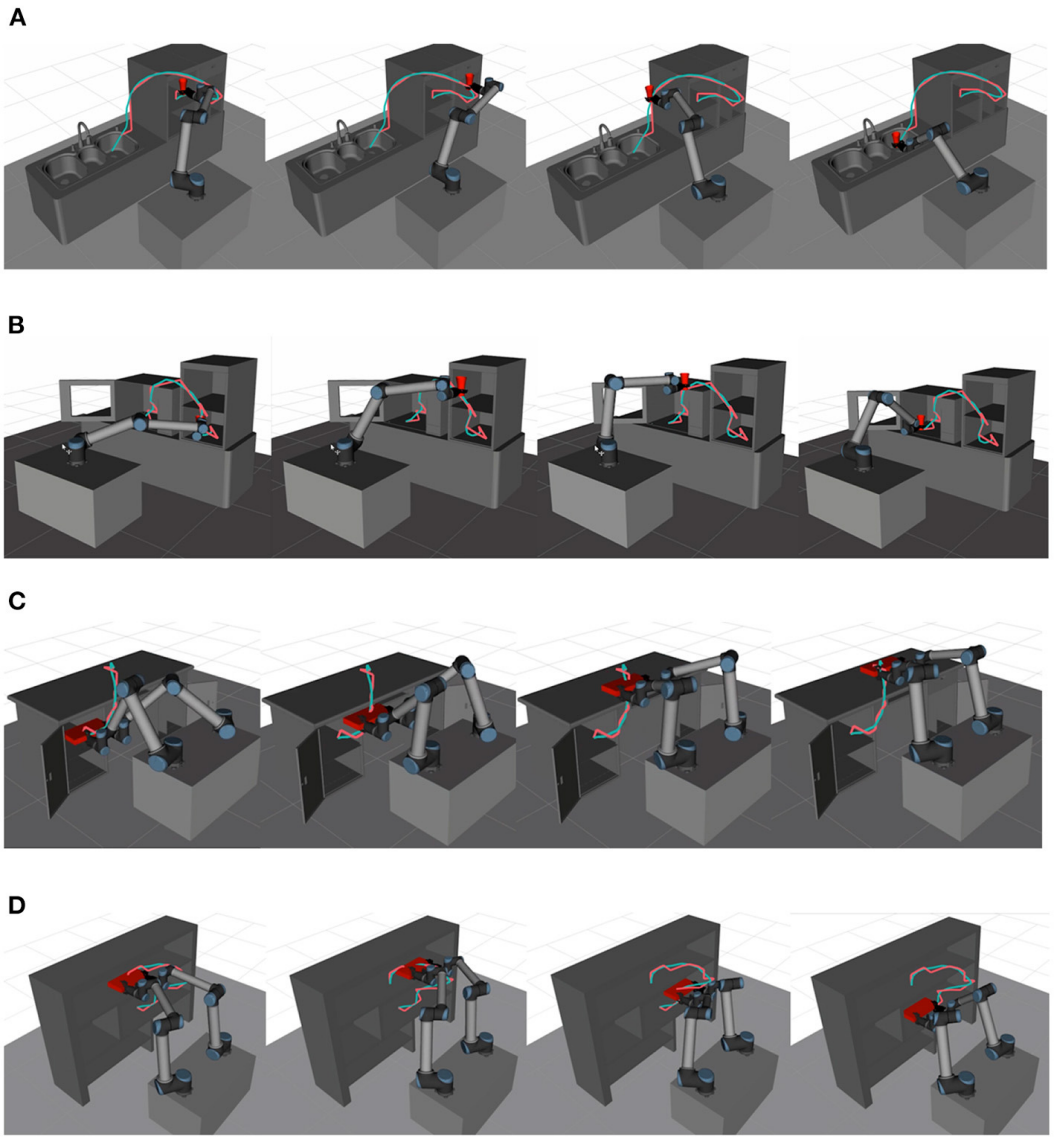
**(A)** A single-arm robot transfers a cup of water in Scene 1; **(B)** A single-arm robot transfers a cup of water in Scene 2; **(C)** A dual-arm robot transfers a pallet in Scene 3; **(D)** A dual-arm robot transfers a pallet in Scene 4. The snapshots of robots performing task constrained trajectories which are optimized by LCQP in different family scenes.

A typical task constraint is a 6-DOF robot holding a cup and keeping it upright during transfer, as shown in Figures 9A,B. Pitch and roll are restricted in this task, which makes the feasible configurations form a continuous four-dimensional manifold in C-space. In Figures 9C,D, two end-effectors controlled by one upper computer work together to maintain a pallet's levelness, and the upper computer tries to optimize the trajectory of the pallet. In this case, the dual-arm robot forms a kinematic closed chain. The configurations that satisfy the constraint constitute a continuous four-dimensional manifold in a 12-dimensional C-space.

To solve the above task constraints, we use Approximate Graph-based Constrained Bi-direction Rapidly-Exploring Tree (AG-CBiRRT) (Zha et al., 2018) which is also a SBMP to plan the original trajectory. AG-CBiRRT improves upon Constrained Bi-direction Rapidly-Exploring Tree (CBiRRT) (Berenson et al., 2011) which can solve various task constraints stably and quickly by manifold metric learning. The update rate α is an empirical value obtained by multiple tests, which is set to 0.2 in all experiments. The step length λ is set to 0.2 and 0.4 rad for a single-arm scenario and a dual-arm scenario, respectively. The terminal condition and robot parameters are the same as 4.2, but the simplification process based on random Short-cut (Line 1 of Algorithm 1) before QP iteration will be not executed.

The snapshots of robots performing task constrained trajectories which are optimized by LCQP are shown in Figure 9. Since the trajectory of robots' high-dimensional C-space cannot be displayed, we use the trajectories of the operated object to express the optimization effect. Intuitively, LCPQ optimization removes a lot of redundant motions in the trajectories, and makes them smoother and more natural. Besides, LCQP optimization enables the robot to meet the task constraints at any moment in the motion process.

As shown in Table 4, the performance comparison between the optimized trajectories and the original ones under the four planning scenarios are obtained through 50 repeated tests. μ and σ are the mean value and the standard deviation of each variable, respectively. The execution time of the LCQP optimized trajectory is significantly shorter than the original trajectory, which can make up for the calculation time overheads for LCQP to some extent. Besides, LCQP removes most of unnecessary acceleration and deceleration, which makes the smoothness ratio of the optimized trajectories closer to 1. *T*_*E*_ and *R* have smaller standard deviation, which means the optimized trajectory has better quality consistency.

**Table 4:** Experimental results of robot motion planning under task constraints.

	***T***_*****P*****_ **(s)**		***T***_*****E*****_ **(s)**		***R***	
	**μ**	**σ**	**μ**	**σ**	**μ**	**σ**
**Scene 1 (Single-arm)**						
AG-CBiRRT Only	0.641	0.151	10.68	1.638	4.560	0.485
AG-CBiRRT + LCQP	4.310	1.061	6.505	1.235	3.307	0.319
**Scene 2 (Single-arm)**						
AG-CBiRRT Only	0.675	0.161	12.89	2.106	4.578	0.427
AG-CBiRRT + LCQP	6.012	1.281	7.346	1.369	2.812	0.321
**Scene 3 (Dual-arm)**						
AG-CBiRRT Only	17.34	5.786	10.92	3.527	4.283	0.475
AG-CBiRRT + LCQP	22.49	6.293	7.677	2.971	3.243	0.423
**Scene 4 (Dual-arm)**						
AG-CBiRRT Only	0.655	0.124	11.65	1.370	4.411	0.346
AG-CBiRRT + LCQP	5.962	1.804	8.992	1.063	3.219	0.297

A series of experiments prove that LCQP can significantly smooth the trajectories and improve the quality consistency while ensuring that the task constraints are met.

### 4.4. Optimization for Passive Chain Constrained Tasks

When a robot manipulates objects with passive chain constraints like doors, drawers or laptops, the actual trajectory of the end effector needs to exactly match the movements of the moving part, which is a stricter task constraint (The end effector and the moving part of the object are considered to be rigidly connected during manipulation). To solve this problem, we assume that the robot and the object are connected by a virtual spring damper, which is implemented by an admittance controller at the algorithm level:

(30) $$\begin{array}{l} BẊ+K\left(X-X_{r}\right)=F_{ext} \end{array}$$

where ***F***_***ext***_ is the external force on the end effector, which can be obtained by a six-dimensional force/torque sensor and some post-processing processes, such as filtering, gravity compensation, dead zone and saturation. ***K*** is a stiffness matrix. ***X***_***r***_ is the reference position which is an interpolation of the trajectory generated by the motion planner, and ***X*** is the target position generated by the admittance controller.

**Figure 10:**
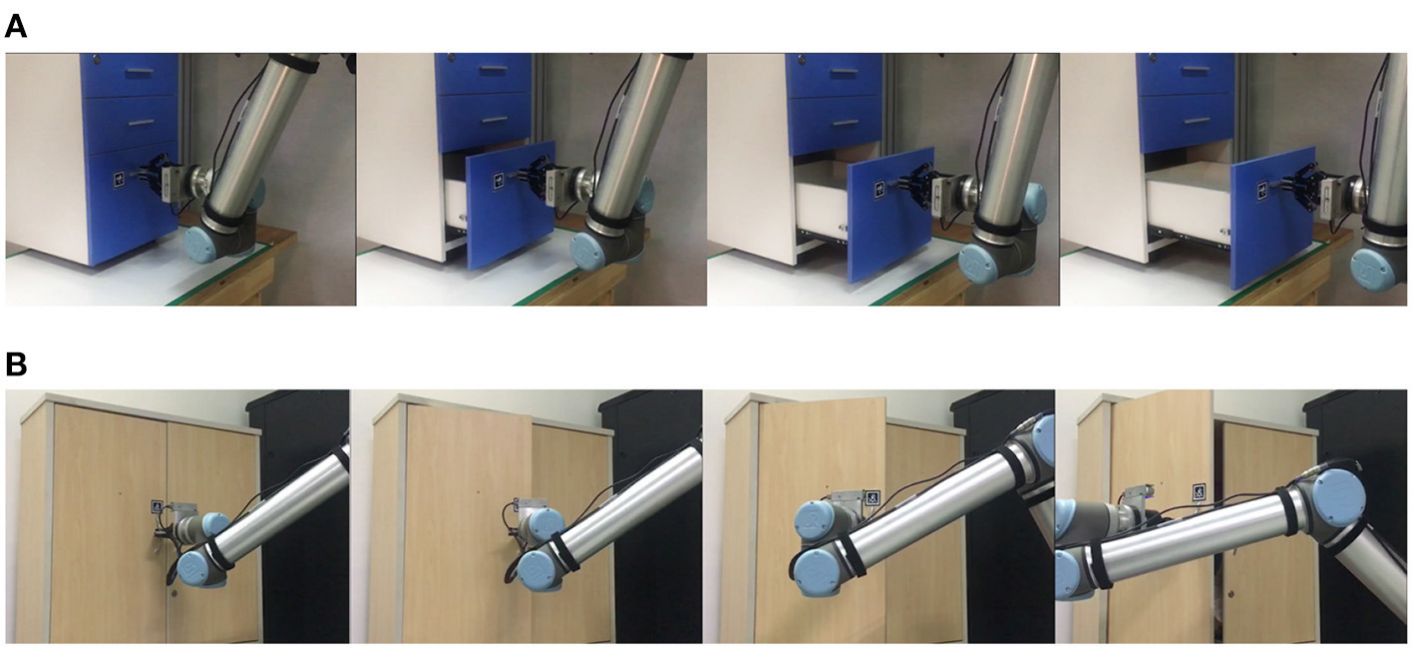
**(A)** Opening the drawer; **(B)** Opening the door. The robot performs the manipulation task under the passive chain constraints.

We test the actual effect of AG-CBiRRT generated trajectories and LCQP optimized trajectories on these passive chain constrained tasks, respectively. As shown in Figure 10, because of the admittance controller, the geometry of the two trajectories is almost the same when they are actually executed, but the force/torque sensor data can still reflect the difference of the trajectories' quality.

**Figure 11:**
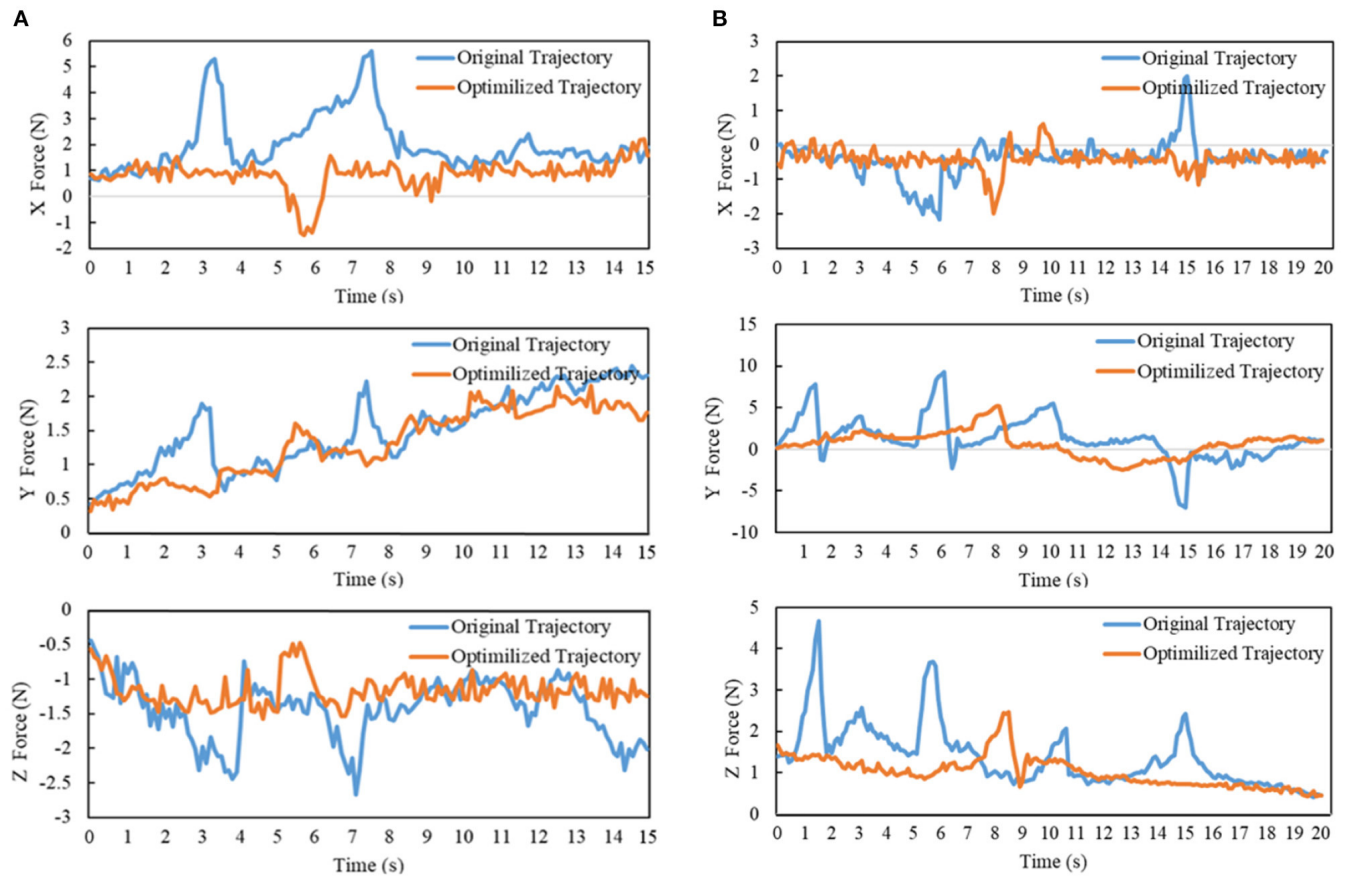
**(A)** The force value of opening drawer process; **(B)** The force value of opening door process. The output force values in three directions of Force/Torque sensor after gravity compensation.

During the above manipulation processes, the force values in X, Y, and Z directions under Force/Torque sensor frame are shown in Figure 11. In order to avoid unnecessary vibration, the admittance controller is used to compensate the end-effector's position only when the force exceeds the dead zone threshold. By observing the change of forces which are perpendicular to the movement direction of the end-effector (X Force in Figure 11A and Y Force in Figure 11B), the LCQP optimized trajectories have less violation to the closed chain constraints, which show a more stable force change. Besides, the forces in the direction of the end-effector's movement (Z Force in Figures 11A,B) also reflect the optimized trajectory can be executed more smoothly than the original trajectory. The above experiments show that LCQP optimization can not only remove the unnecessary accelerations, but also make the trajectories better meet the task constraints through repeated manifold projections.

## 5. Conclusion

This paper introduces a trajectory post-process method that uses linearly constrained quadratic programming to transform a randomly polygonal collision-free trajectory produced by SBMPs into a smoother one. This method combines the advantages of GBO and Short-cut. Firstly, we use randomly Short-cut to remove redundant motions in initial trajectory. Then, quadratic programming is used to optimize the trajectory in an infinite dimensional parameter space by a smooth gradient function. We check the feasible state of the trajectory after each optimization update. If a collision occurs, a linear constraint will be added to ensure there is no more collision at this position in future optimization, and the trajectory is backtracked to the previous state. This mechanism ensures the security of the optimized trajectory. Furthermore, we extend the method to task constrained motion planning, which requires the trajectory to be located on the surface of a nonlinear low-dimensional manifold. The projection technique is used to ensure the optimized trajectory always satisfies the task constraints. Finally, we introduce two quantitative indicators to evaluate path quality, and conduct a series of experiments for comparison with the state-of-the-art trajectory post-process methods. The experimental results show the proposed methods can significantly improve the trajectory quality on the basis of satisfying environment and task constraints.

However, LCQP is a gradient-based numerical optimization method, which needs more time overheads compared with SBMPs and Short-Cuts. This will be a bottleneck in practical applications. So in the future, we will improve the LCQP efficiency by parallel computation and GPU acceleration, and try to reduce the time overheads to the same order of magnitude as SBMPs. Besides, we will apply the LCQP to more robot platforms, especially legged robots, and test the robustness of the algorithm in various complex scenarios.

## Data Availability Statement

The original contributions presented in the study are included in the article/supplementary material, further inquiries can be directed to the corresponding author/s.

## Author Contributions

YL made substantial contributions to the original ideas, designed the experiments, and wrote the manuscript. FZ developed the simulation platform and performed the experiments. YZ typeset the manuscript and is accountable for the publishing issues. ML, PW, and YJ provide financial support for the study. LS supervised, analyzed the results, provided feedback, and revised the manuscript. All authors contributed to the article and approved the submitted version.

## Conflict of Interest

YJ is employed by Harbin Mingkuai Machinery & Electronics Co., Ltd. The remaining authors declare that the research was conducted in the absence of any commercial or financial relationships that could be construed as a potential conflict of interest.

## Publisher's Note

All claims expressed in this article are solely those of the authors and do not necessarily represent those of their affiliated organizations, or those of the publisher, the editors and the reviewers. Any product that may be evaluated in this article, or claim that may be made by its manufacturer, is not guaranteed or endorsed by the publisher.
